# Morphostructural studies of pure and mixed metal oxide nanoparticles of Cu with Ni and Zn

**DOI:** 10.1016/j.heliyon.2024.e30544

**Published:** 2024-05-01

**Authors:** Md. Jasim Uddin, Mst. Sarmina Yeasmin, Ali Ahsan Muzahid, Md. Mahmudur Rahman, G.M. Masud Rana, Tahmina Akter Chowdhury, Md. Al-Amin, Md. Kazi Wakib, Sayeda Halima Begum

**Affiliations:** a Bangladesh Council of Scientific and Industrial Research (BCSIR), Rajshahi Laboratories, Rajshahi, 6206, Bangladesh; b Department of Chemistry, University of Chittagong, Chittagong, 4331, Bangladesh

**Keywords:** CuO, CuO–NiO, CuO–NiO–ZnO nanoparticles, Characterization, Morphostructural properties

## Abstract

Nano-scale interactions between pure metal or metal-oxide components within an oxide matrix can improve functional performance over basic metal oxides. This study reports on the synthesis of monometallic (CuO), bimetallic (CuO–NiO) and trimetallic (CuO–NiO–ZnO) oxide nanoparticles (NPs) via the co-precipitation method and investigation of morphostructural properties. All of the synthesized metal oxide NPs were calcined at 550 °C temperature and annealed under vacuum. In this work, we applied Scherrer formula, modified Scherrer equation, Williamson-Hall plots, and Halder-Wagner plots to calculate the average crystallite size. The XRD data analysis showed that average crystallite sizes of the as-synthesized metal oxide phases were between 4 nm and 76 nm and average diameters calculated from SEM image were between 15 nm and 83 nm. The XRD studies also disclosed that average crystallite size and lattice microstrain of the CuO phases remain almost same (43 nm–46 nm and $2.074\times 10^{-3}$ to $2.665\times 10^{-3}$) for pure CuO and mixed CuO–NiO; but in case of mixed CuO–NiO–ZnO it is found to decrease in size to 11 nm where lattice microstrain increases to $9.653\times 10^{-3}$. Line broadening of diffraction peaks from microstrain contribution was between 0.02 and 0.01. Degree of crystallinity (%) of CuO phases found to decrease from 81 to 71. Dislocation density of CuO phases found to increase from $6.63\times 10^{-4}\;{nm}^{-2}$ to $12.68\times 10^{-3}\;{nm}^{-2}$. X-ray density of CuO phases increased from 6.48 to 6.53 g/cm^3^. Where this calculated small dislocation density well agreed with the high crystallinity. Crystal structure and specific surface area were determined from lattice constants and X-ray density. These synthesized nanopowders showed the existence of monoclinic, cubic, and hexagonal phases. The obtained NPs of multi-metal oxide explained more than one phases with different size, shape, and morphology at nano scale.

## 1 Introduction

Nanomaterials of pure/multi metal oxides, have emerged as pivotal components in chemistry and materials science. These nanoscale structures possess distinctive physical properties, making them valuable in various industrial applications such as biomedicine, microelectronics, sensors, fuel cells, and corrosion protection coatings etc. [1]. In the realm of nanotechnology, there has been a growing research emphasis on nanostructured metal oxides composed of two or more distinct metallic elements [2], showcasing their distinct electronic, optical, magnetic, and other physicochemical attributes. Nanocomposites of multi-metal oxide like Zn_x_Mg_1−x_O, Ta-doped ZnO, Ag/Fe_3_O_4_ and NiO–CdO–ZnO nanocomposites are being studied as potential antimicrobial and photocatalytic agents which overcome high cytotoxicity or agglomeration seen in pure metal oxide nanoparticles [3,4]. Nanomanufacturing means the production of improved materials and novel products at nanoscale. Which involves scaled-up, reliable and cost-effective manufacturing [5]. A multitude of materials are being generated by manipulating visible objects on a larger scale, while nanotechnology represents a cutting-edge avenue enabling researchers to manipulate the fundamental building blocks of matter. This offers a novel pathway towards creating new materials with unprecedented properties [6]. The characteristics of nanomaterials are influenced by both their composition and the sizes and shapes of particles [7]. By altering the nanoparticle size, composition, crystal structure and morphology these properties can be adjusted [8]. A recent study demonstrates the influence of calcination temperature on the characteristic properties of metal-oxide NPs [9]. Ensuring uniform particle sizes is crucial for optical applications of NPs. To achieve this, stabilizing the particle surface with a dispersing agent is necessary. Various studies have investigated dispersing agents to maintain high nanoparticle dispersity. According to the Hard and Soft Acids and Bases (HSAB) rule; Ag^+^, Au^+^, Pd^2+^, Pt^2+^ are classified as soft acids [10] and substrates processing the thiol (R–SH) and the phosphine (P-R3) functional groups, classified as soft bases, have demonstrated effectiveness as dispersing agents. Early investigations into the use of organic thiol molecules as potential dispersants were reported by Brust and co-workers [11]. Various methods can be employed to synthesize metal-oxide NPs, including chemical reduction, photochemical processes, electroreduction, electro-exploding, microemulsion, gamma radiolysis, sonochemical reduction, co-precipitation, metal vapor deposition, and microwave irradiation etc. [12]. In the co-precipitation method, the straightforward synthesis entails precipitating the oxo-hydroxide form from a solution containing a salt precursor in a solvent, such as water, utilizing a precipitating medium [13]. Using various bases such as NaOH, KOH, NH_4_OH, and (C_2_H_5_OH) as precipitation agents has been demonstrated to influence the size, shape and level of agglomeration of synthesized NPs. Factors such as pH, type of alkali, gradual or rapid addition of alkaline solution, and drying method contribute to these effects. This approach presents advantages such as cost-effectiveness, favorable reaction conditions (including reduced synthesis temperatures and the potential for direct water synthesis), straightforward processing, scalability, and the capacity to tailor core and surface properties. It has been observed that pure and mixed oxide NPs of metals like Cu, Ni and Zn have gained huge attraction towards researchers. Copper oxide NPs possess advantageous properties like direct band gap, intrinsic p-type behavior, cost-effective production, and strong electrochemical performance. Recent findings highlight enhanced thermal conductivity in nanofluids composed of CuO or Al_2_O_3_ NPs in water or ethylene, showcasing advanced thermal properties. Copper oxide NPs demonstrate remarkable thermal conductivity, surpassing water by approximately 700 times and engine oil by 3000 times at room temperature [14]. Within the copper oxide system, cupric oxide (CuO) and cuprous oxide (Cu_2_O) hold significance. Monoclinic CuO-NPs are utilized as antioxidants [15], antibacterial agents, and catalysts [16]. In a recent work, copper sulphide nanoparticles were studied to improve solar energy harvesting and found enhanced photovoltaic parameters [17]. Nickel Oxide (NiO) exists in two forms rhombohedral and cubic; black, and green in color, respectively. Cubic rock salt structure is stoichiometric and other one is non-stoichiometric in nature. Nickel oxide has interesting applications such as hydrogen storage and catalytic property [18]. Impact of yttrium doping on dislocation density, lattice strain and surface area for Nickel Oxide was explained for semiconductor applications [19]. NiO and CuO exhibit optical band gaps of 3.6–4eV and 1.2eV [20], respectively. Both serve as P-type semiconductors, offering versatile applications in sensors, optical devices, supercapacitors, transparent semiconducting layers, and antiferromagnetic films [21]. Metal oxides nanostructures, like CuO–NiO nanocomposites, offer distinctive magnetic, optical and molecular properties arising from the mixing of NiO and CuO phases. These composed materials find applications in various fields, including catalytic activity [22], antibacterial and antifungal activity [23], nano-biosensor, drug delivery, immunological assays [[24], [25], [26]] etc. Nanocomposites of nickel-zinc have been reported to exhibit antifungal properties against *Penicillium chrysogenum* fungi [26]. ZnO NPs, characterized by a high band gap (∼3.3 eV) and an exciton binding energy of around 60 meV [27] have various optoelectrical applications. A recent study assessed the photocatalytic and bioactivity of ZnO-NPs [28]. Plasmonic metal NPs, such as Ag:Zn bimetallic nanocomposites can exhibit enhanced optical and electrical properties due to their ability to concentrate and manipulate electromagnetic fields at the nanoscale [29]. In a recent work a multiphase CuO–NiO–ZnO mixed ternary oxide nanocomposites was synthesized by a homogenous precipitation method and the observed band gap 1.68 eV falls between those of CuO, ZnO and NiO [30]. Some other studies uncovered the potential activity of ternary oxide nanocomposites of Cu, Ni and Zn as antibacterial [31] agent, and as smart electrode material [32]. With all of this in consider, in the current study, the CuO, CuO–NiO and CuO–NiO–ZnO nanometer-sized powders were synthesized via simple co-precipitation method and their important morphostructural characteristics were investigated by FTIR, XRD, SEM and EDX studies. In this work, we have used ammonium carbonate as a reducing agent, a specific gradient solvent of water and alcohol with a ratio of 9:2 as a stabilizing agent, and finally, all the synthesized nanopowders were calcined at 550 °C. Tuning the synthesis parameters and calcination temperature, it is possible to modify the properties of NPs to meet the demands of particular applications. So, one of the significant novelties of this work lies in the controlled synthesis of these pure and mixed metal oxide nanoparticles comprising Cu, Ni, and Zn. As optoelectrical properties of metal oxide NPs depend on their shape and size, the presence of phase difference of NiO in CuO–NiO (ditrigonal scalenohedral) and CuO–NiO–ZnO (hexoctahedral) is another novelty. By combining these metal oxides into mixed NPs, the study aimed to explore synergistic effects [33] and tunable properties. The focus of the study was to investigate the morphostructural properties of pure and mixed metal oxide nanoparticles composed of copper (Cu) with nickel (Ni) and zinc (Zn).

## 2 Materials and methods

### 2.1 Synthesis

#### 2.1.1 Synthesis of CuO monometallic NPs, CuO–NiO bimetallic NPs and CuO–NiO–ZnO trimetallic NPs

In the preparation of CuO, CuO–NiO and CuO–NiO–ZnO NPs analytical grade of copper (II) nitrate trihydrate (Cu (NO_3_)_2_·3H_2_O) (Merck, Germany) as the Cu salt, nickel nitrate hexahydrate (Ni(NO_3_)_2_·6H_2_O) (Merck, Germany) as the Ni salt, zinc nitrate hexahydrate (Zn(NO_3_)_2_·6H_2_O) (Merck, Germany) as the Zn salt, and ammonium carbonate were used.

To synthesis the CuO NPs, a solution containing 1.208 g of Cu salt was prepared in a solvent mixture of water and alcohol (in a 9:2 vol ratio). This solution was stirred magnetically at room temperature for 20 min. Separately, an aqueous solution (110 mL) of (NH_4_)_2_CO_3_ was prepared by dissolving 0.5 g of (NH_4_)_2_CO_3_ salt in double distilled water and stirring for 15 min. Then in the course of magnetically stirred of copper nitrate solution, the ammonium carbonate solution was added dropwise as a precursor agent at 80 °C for 1 h, resulting in light green sediments. These precipitates were filtered and washed with distilled water for several times. The washed precipitates were dried in oven at certain time to get the precursors. Pure CuO was obtained by annealing these precursors at 550 °C in muffle furnace under vacuum for 2 h to turn into black powder.

For the synthesis of CuO–NiO NPs, a solution containing 1.208 g of Cu salt and 1.454 g Ni salt with Cu:Ni mole ratio of 1:1 was prepared in a solvent mixture of water and alcohol (in a 9:2 vol ratio). And for the synthesis of CuO–NiO–ZnO NPs, a solution containing 1.208 g of Cu salt 1.454 g of Ni salt and 1.4875 g of Zn salt with Cu:Ni:Zn mole ratio of 1:1:1 was prepared. Similar steps were undertaken, which involved preparing solutions, adding ammonium carbonate as a precursor, filtering, washing, and drying the resulting precipitates. Finally, annealing these precursors at 550 °C in a vacuum furnace yielded the desired pure nanoparticles. The chemical reactions can be followed as:

[Cu (NO_3_)_2_.3H_2_O] + (NH_4_)_2_CO_3_ CuCO_3_(OH)_2_ + 2NH_4_NO_3_

CuCO_3_(OH)_2_ CuO

[Cu (NO_3_)_2_.3H_2_O] + [Ni (NO_3_)_2_.6H_2_O] + 2(NH_4_)_2_CO_3_ CuCO_3_(OH)_2_– Ni_4_CO_3_(OH)_6_.4(H_2_O) + 4NH_4_NO_3_

CuCO_3_(OH)_2_– Ni_4_CO_3_(OH)_6_.4(H_2_O) CuO–NiO

Zn (NO_3_)_2_.6H_2_O] + [Ni (NO_3_)_2_.6H_2_O] + [Cu (NO_3_)_2_·3H_2_O] + 3 (NH_4_)_2_CO_3_ CuCO_3_(OH)_2_– Ni_4_CO_3_(OH)_6_.4(H_2_O)– ZnCO_3_(OH)_6_.4(H_2_O) + 6NH_4_NO_3_

CuCO_3_(OH)_2_– Ni_4_CO_3_(OH)_6_.4(H_2_O)– ZnCO_3_(OH)_6_.4(H_2_O) CuO–NiO–ZnO

The synthesis method showcased an effortless production of these NPs laying valuable insights for future research and applications in nanotechnology and materials science.

### 2.2 Characterization techniques

#### 2.2.1 Fourier Transform infrared (FT-IR)

Fourier Transform infrared spectroscopy encompasses the interaction of infrared radiation with matter which mostly based on adsorption spectroscopy. All spectra were obtained within 400–4000 cm^−1^ with 20 scans per measurement at 0.4 cm^−1^ resolution with the FTIR apparatus (Shimadzu IR Prestige-21 spectrometer). Sample powders were mixed with dry KBr powder and compressed into pellets using a hydraulic press. Maintaining a consistent compression magnitude is crucial to ensure uniform absorbance across samples. Spectra of CuO, CuO–NiO, and CuO–NiO–ZnO mixed powders were then obtained. Nanoparticles, with their high surface area to volume ratio, have the ability to absorb moisture from their surroundings [34]. The absorption band at 1615∼1640 cm^−1^ indicates the bending mode of vibration in water (O–H) group, and the wide and narrow band observe in the band at 3200∼3550 cm^−1^ shows the stretching mode of vibration in hydroxyl group (O–H) [35]. The FTIR spectra of copper oxide, copper-nickel, and copper-nickel-zinc mixed oxides were analyzed and compared to those of the corresponding nano oxides. In nano-sized grains, the atomic arrangement at boundaries significantly differs from that of bulk crystals, exhibiting some disorder in coordination number and bond lengths. Consequently, crystal symmetry is diminished in nano-sized grains, leading to a shift in the IR active mode [36].

#### 2.2.2 X-ray diffraction (XRD)

Characterization of synthesized CuO, CuO–NiO and CuO–NiO–ZnO mixed oxide NPs were studied to analyze the phase. X-ray diffraction peaks were produced by constructive interference of a monochromatic beam of X-rays diffracted at specific angles from each set of lattice planes in a sample. The XRD patterns with diffraction intensity versus 2 θ were recorded by XRD instrument (BRUKER D8 ADVANCE wide-angle X-ray diffractometer) that possesses a voltage of 50 kV plus current of 40 mA and Cu Kα radiation (*α* = 0.154 nm). Dislocation density, average crystallite size, microstrain, stress, the X-ray density of crystal, specific surface area, crystallite size distribution and morphologies of the crystals were determined based on X-ray diffraction profile analysis.

#### 2.2.3 Scanning electron microscopy (SEM)

A Scanning Electron Microscope (SEM) generates images by scanning a focused electron over a surface. Interactions between the electrons and the sample producing various signals revealing details about surface topography and composition. It provides a pseudo 3-dimensional reconstruction of the sample based on signals emitted during electron-sample interactions [37]. The morphologies of synthesized CuO, CuO–NiO and CuO–NiO–ZnO mixed oxide NPs were analyzed by SEM instrumental setup with a model number of JSM-7610 F. The accelerating voltage was maintained by 10–20 kV with a variety of magnifications. The images are shown as in the following pages.

#### 2.2.4 Energy dispersive X-ray (EDX)

Energy dispersive X-ray (EDX) spectroscopy is an analytical method employed for elemental analysis, chemical characterization, and determining relative abundances within a sample. However, the accuracy of this quantitative sample composition analysis is influenced by several factors. X-rays are produced when any atom in the sample is adequately excited by the incoming beam. These X-rays are emitted in all directions (isotropically), but not all may escape the sample. The likelihood of an X-ray escaping and being available for detection and measurement depends on its energy, as well as the composition, quantity, and density of material it traverses to reach the detector. Due to X-ray absorption and similar phenomena, precise estimation of sample composition from the measured X-ray emission spectrum necessitates the application of quantitative correction procedures, often known as matrix corrections. Many elements may have overlapping X-ray emission peaks (e.g., Ti-K_β_ and V-K_α_, Mn-K_β_ and Fe-K_α_) [38]. The elemental analysis of synthesized CuO, CuO–NiO and CuO–NiO–ZnO mixed oxide NPs were analyzed by EDX which was associated with SEM instrumental setup with a model number of JSM-7610 F. Results are shown as in the following pages.

## 3 Results and discussion

### 3.1 Fourier Transform infrared (FT-IR) analysis

**Fig. 1:**
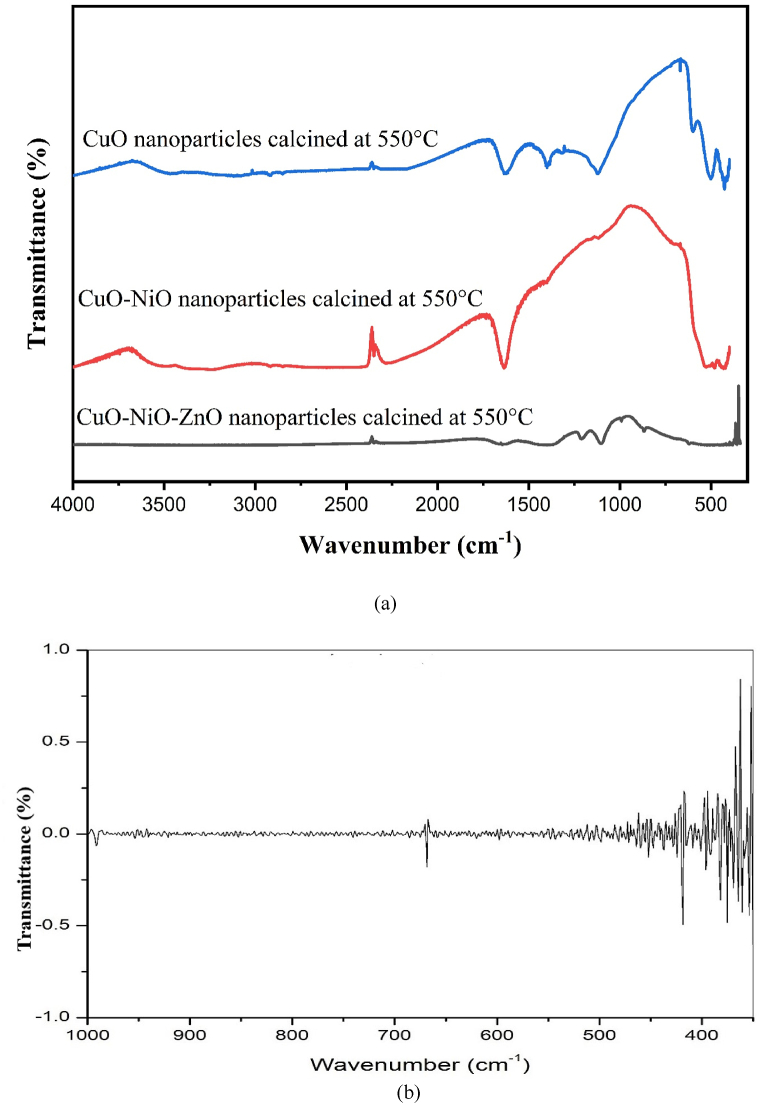
The FTIR spectrum of nano-powders annealed at 550 °C denoted as (a) for CuO, CuO–NiO and CuO–NiO–ZnO nano-powders and (b) for CuO–NiO–ZnO nano-powder which indicates the overlapped vibration peaks occurring in the region below 600 cm^−1^ for CuO, NiO and ZnO.

The vibrational frequency was confirmed by FT-IR analysis where O–H stretching vibration mode showed absorption spectrum at 3350–3430 cm^−1^ and band near 1630–1635 cm^−1^ for H–OH bending vibration mode. It was due to adsorption of moisture/water onto the surface during sampling. Bands found at the region of 1000–1500 cm^−1^ for O^−^, C–O stretching vibration [39]. It was found the peak at 439.98 cm^−1^, 497.55 cm^−1^ and 593.51 cm^−1^ indicating the formation of the CuO NPs [40], band at 511.17 cm^−1^ & 546.08 cm^−1^ indicating the presence of CuO mixed with NiO NPs [41,42] and overlapping vibration peaks occurred in the region below 600 cm^−1^ for CuO, NiO and ZnO NPs; as shown in Fig. 1 (a). The FTIR spectrum of the CuO–NiO–ZnO NPs is given in Fig. 1 (b). The proximity of their absorption peaks complicates the identification of distinct peaks for each oxide phase. CuO has infrared vibrational bands at 478.89 cm^−1^, 619.78 cm^−1^, and 592.20 cm^−1^ [43]. The vibrational bands for the Zn–O bond occurs at 396.25 cm^−1^, 425.66 cm^−1^ and 473.01 cm^−1^ depending on the shape morphology of the NPs [44,45]. NiO shows IR absorption peaks due to Ni–O bond vibration at 456.65 cm^−1^, 431.32 cm^−1^ and 570.60 cm^−1^ [46]. In Fig. 1(b) the observed broad bands occurring below 1000 cm^−1^ are for metal-oxygen bond vibrations.

### 3.2 The X-ray diffraction (XRD) patterns analysis

#### 3.2.1 The X-ray diffraction patterns analysis of CuO NPs

**Fig. 2:**
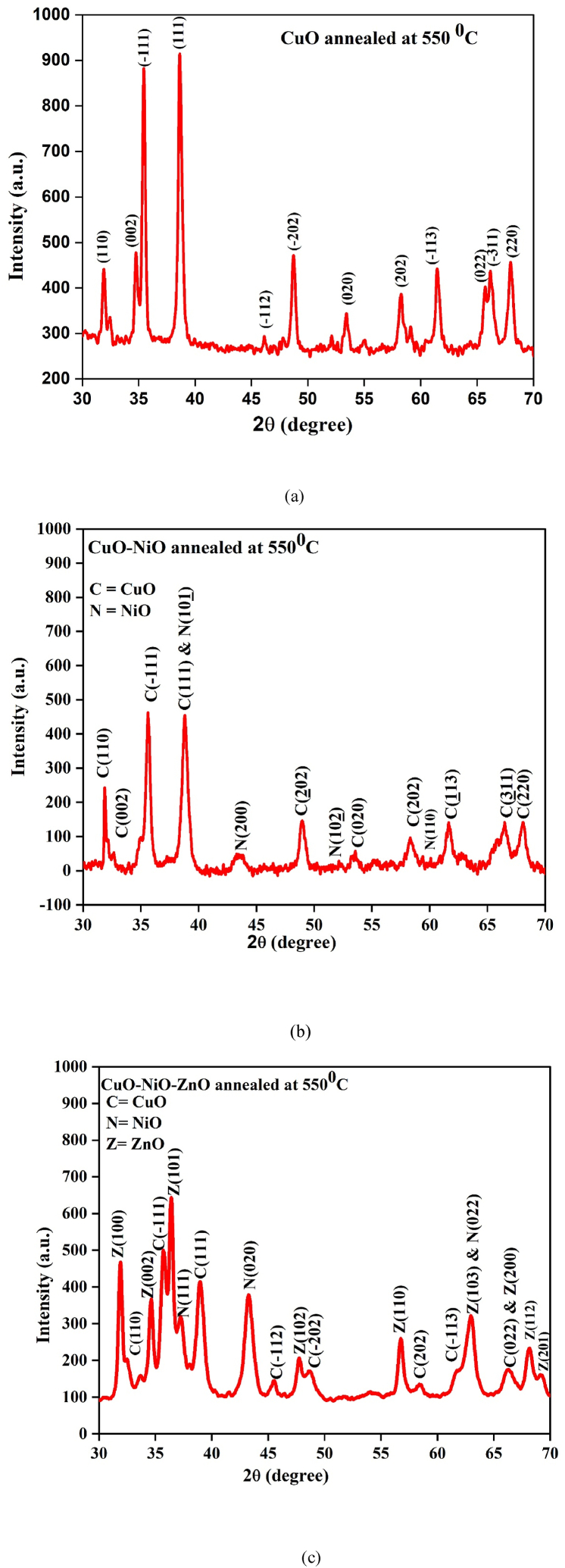
The XRD diffractograms denoted as (a), (b) and (c) for CuO, CuO–NiO and CuO–NiO–ZnO nano-powders annealed at 550 °C, respectively.

The XRD patterns of as-synthesized CuO NPs after annealing at 550 °C is shown in Fig. 2 (a). XRD peaks confirm that the formation of CuO from each precursor was in monoclinic phase. The characteristic peaks located at 2 θ = 31.9219°, 35.4576°, 38.5973°, 48.7430°, 53.4226°, 58.3025°, 61.4408°, 66.2459° and 67.9836° which corresponds to (110), ($\bar{1}$ 11), (111), ($\bar{2}$ 02), (020), (202), ($\bar{1}$ 13), ($\bar{3}$ 11) and (220) planes, respectively, indicated the formation of a typical monoclinic CuO NPs structure without impurities. Sharp, well-defined CuO reflections in the XRD pattern confirmed the highly crystalline nature as synthesized CuO NPs. The recorded XRD pattern of this sample agrees well with the JCPDS file (JCPDS Card No. 96-901-6327) and results show that the predominant diffraction lines correspond to CuO lattice are at d (Å) = 2.80359, 2.53171, 2.33269, 1.86825, 1.71512, 1.58265, 1.50913, 1.41084 and 1.37895. The recorded XRD pattern of this sample agrees well with the (COD No. 9016326).

#### 3.2.2 The X-ray diffraction patterns analysis of CuO–NiO NPs

The XRD patterns of as-synthesized CuO–NiO NPs after annealing at 550 °C is shown in Fig. 2 (b). The characteristic peaks located at 2 θ = 31.8613°, 35.6089°, 38.7952°, 48.8447°, 53.452°, 58.2902°, 61.6943°, 66.4647° and 68.0757° which corresponds to (110), ($\bar{1}$ 11), (111), ($\bar{2}$ 02), (020), (202) ($\bar{1}$ 13), ($\bar{3}$ 11) and (220) planes, respectively, indicated the formation of a typical monoclinic CuO NPs. The characteristic peaks located at 2θ = 19.301°, 33.161°, 38.654°, 43.274°, 52.243°, 59.242° and 62.921° which corresponds to (001), (100), (10 $\bar{1}$), (200), (10 $\bar{2}$), (110) and (111) planes, respectively, indicated the formation of a typical hexagonal NiO NPs structure without impurities. Sharp, well-defined CuO–NiO mixed reflections in the XRD pattern confirmed the highly crystalline nature as synthesized CuO–NiO mixed NPs. The recorded XRD pattern of this sample agrees well with the JCPDS file (JCPDS Card No. 96-721-2243) and (JCPDS Card No. 96-900-9113). The recorded XRD pattern of this sample agrees well with the (COD No. 7212242) and (COD No. 9009112).

The relative intensity ratio of the characteristic peaks of CuO (R_CuO_) and NiO (R_NiO_) correlates with the volume fractions and can be calculated using the following expressions [47];

(1) $$R_{\text{CuO}}=\frac{I_{\text{CuO}}}{I_{\text{CuO}}+I_{\text{NiO}\;}}\times 100\%$$

(2) $$R_{\text{NiO}}=\frac{I_{\text{NiO}}}{I_{\text{CuO}}+I_{\text{NiO}\;}}\times 100\%$$

where I_CuO_ and I_NiO_ are the characteristic peaks of CuO and NiO; which are (111) and (200), respectively. The calculated values of the relative intensity ratio of R_CuO_ and R_NiO_ by following equations (1), (2) are therefore 87.27 % and 12.73 %, respectively. Selected Miller planes were used to determine crystallite size for CuO phase and NiO phase in CuO–NiO mixed nanopowders [39]. Variability in synthesis parameters can contribute to the observed differences in composition. Small variations in experimental parameters can significantly impact the nucleation, growth, and composition of the resulting nanoparticles [48]. Copper and nickel salts would exhibit differential reactivity during the synthesis conditions we used, leading to preferential nucleation and growth of CuO nanoparticles over NiO nanoparticles. Again, the solubility of copper and nickel salts, as well as their corresponding hydroxides and carbonates, vary under the reaction conditions employed. Variations in solubility might affect the precipitation kinetics [49] and yield of CuO and NiO nanoparticles, leading to unequal incorporation of copper and nickel ions into the final product. Another possibility of copper and nickel ions might exist in different oxidation states and coordination environments in the precursor salts or during the synthesis process. Variations in oxidation state or coordination geometry would influence the chemical reactivity and subsequent formation of CuO and NiO nanoparticles, resulting in non-stoichiometric compositions [50]. CuO and NiO nanoparticles may undergo phase transformations [49] or segregation phenomena during synthesis, leading to the formation of non-uniform compositions.

#### 3.2.3 The X-ray diffraction patterns analysis of CuO–NiO–ZnO NPs

The XRD patterns of as-synthesized CuO–NiO–ZnO NPs after annealing at 550 °C is shown in Fig. 2 (c). The characteristic peaks located at 2 θ = 31.847°, 32.531°, 34.55°, 35.466°, 35.556°, 36.354°, 37.237°, 38.751°, 38.965°, 43.265°, 45.50°, 47.694°, 48.22°, 56.745°, 62.846°, 63.087°, 66.279°, 66.558°, 68.161°, 69.284°, 75.373°, 79.364° and 83.298°. The diffraction peaks appearing at angles 2 θ = 32.531°, 35.466°, 35.556°, 38.751°, 38.965°, 48.751° and 66.279° which corresponds to (110), (002), ($\bar{1}$ 11), (111), (200), ($\bar{2}$ 02) and ($\bar{3}11$) planes, respectively, indicated the formation of a typical monoclinic CuO NPs. The recorded XRD pattern of this sample agrees well with the (COD No. 7212242). The diffraction peaks appearing at angles 2 θ = 37.237°, 43.265°, 62.846°, 75.373° and 79.364° which correspond to the Miller indices (111), (020), (022), (131) and (222) planes, respectively, indicated the formation of the cubic NiO NPs. The recorded XRD pattern of this sample agrees well with the (COD No. 4329323). The diffraction peaks appearing at angles 2 θ = 31.847°, 34.55°, 36.354°, 47.694°, 56.745°, 63.087°, 66.558°, 68.161° and 69.284° which correspond to the Miller indices (100), (002), (101), (102), (110), (103), (200), (112), and (201) planes respectively, indicated the formation of hexagonal ZnO NPs. The recorded XRD pattern of this sample agrees well with the (COD No. 2107059).

Some diffraction peaks overlap with each other since they occur at neighboring diffraction angles for constituent oxides. Therefore, the synthesized nanopowder is a mixture of the individual binary oxide phases co-existing in one material.

**Fig. 3:**
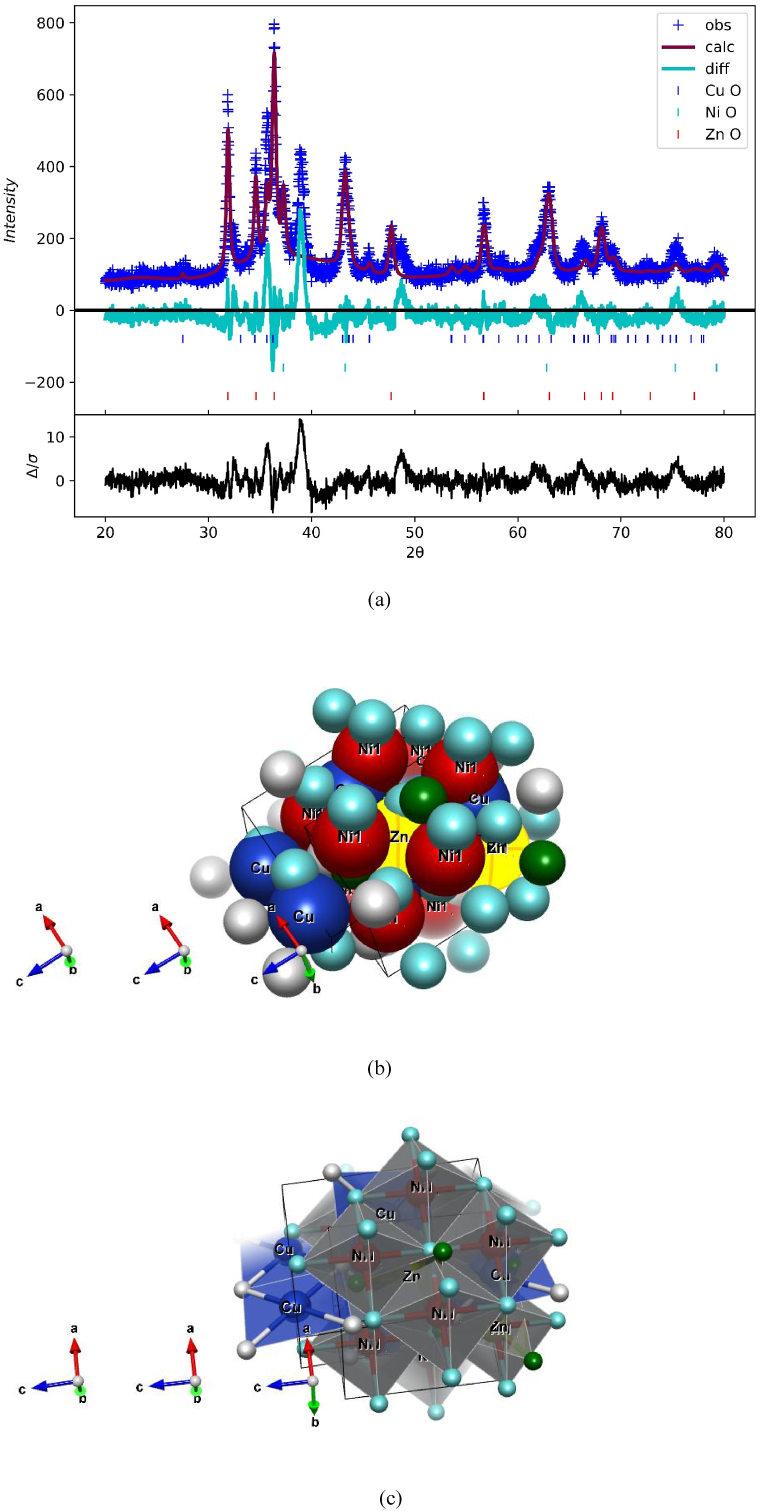
The Rietveld refinement fitting of XRD pattern denoted as (a) and atomic positions denoted as (b) and (c) for CuO–NiO–ZnO nano-powders annealed at 550 °C.

The relative intensity ratio of the characteristic peaks of CuO (R_CuO_), NiO (R_NiO_) and ZnO (R_ZnO_) with the volume fractions and can be calculated using the following expressions [47];

(3) $$R_{\text{CuO}}=\frac{I_{\text{CuO}}}{I_{\text{CuO}}+I_{\text{NiO}\;}+I_{\text{ZnO}}}\times 100\%$$

(4) $$R_{\text{NiO}}=\frac{I_{\text{NiO}}}{I_{\text{CuO}}+I_{\text{NiO}\;}+I_{\text{ZnO}}}\times 100\%$$

(5) $$R_{\text{ZnO}}=\frac{I_{\text{ZnO}}}{I_{\text{CuO}}+I_{\text{NiO}\;}+I_{\text{ZnO}}}\times 100\%$$

where I_CuO_, I_NiO,_ and I_ZnO_, are the characteristic peaks of CuO, NiO and ZnO and which are (111), (020) and (101), respectively. The relative intensity ratios obtained from equations (3), (4), (5) for R_CuO_, R_NiO_ and R_ZnO_ are 23.785 %, 23.062 % and 53.152 %, respectively. The highest volume fraction is obtained for ZnO and the least for NiO. The difference in the volume fractions can be related to the difference in the NPs precipitation yield among the oxides. The Rietveld refinement method was also studied for the sample CuO–NiO–ZnO nanopowder to find the phase fraction and atomic positions shown in Fig. 3 (a), (b) and (c). The results of the studied showed that three phases present as CuO with phase fraction 38.6 %, NiO with phase fraction 36.9 % and ZnO with phase fraction 24.5 %.

#### 3.2.4 Determination of the average crystallite size and the lattice microstrain

Crystallite is a small crystal which could be made of atoms/molecules. Simply, a crystallite is that portion of crystal from which light diffracts coherently. In general, crystallite size is smaller than grain size and the grain size is smaller than particle size. But, in case of NPs grain and crystallite sizes are same. The crystallite size (D) and microstrain contribute to the line broadening in X-ray diffraction [51]. The contribution from the average crystallite size has been estimated by using Scherrer formula [52];

(6) $$D=\frac{K\;ʎ}{\beta_{D}\;\text{COS}\theta }$$

Where k = 0.9 is the shape factor; λ is wavelength of X-ray (0·1541 nm); $\beta_{D}$ is FWHM (full width at half maximum) related to instrument broadening; θ is diffraction angle; D is particle diameter size; $\beta_{S\;}$ is the FWHM linked to the line broadening arising from microstrain, and Ɛ is the root mean square (RMS) value of lattice microstrain, assumed uniform across all crystallographic directions.

For comparison study, the average crystallite sizes were also calculated by applying the modified Scherrer equation and Williamson-Hall (W–H) plots.

Now, modified Scherrer equation is written from equation (4) as followed;

(7) $$\ln\left(\beta \right)=\ln\frac{1}{\cos\;\theta }+\ln\;\frac{K\lambda }{D}$$

By plotting ln (β) against ln $\frac{1}{\cos\;\theta }$ for CuO, NiO and ZnO; the average crystal size was obtained from the y-intercept.

The lattice microstrain contribution is given by Ref. [34];

(8) $$\beta_{Ɛ}=4\;Ɛ\;\tan\;Ɵ$$

And the broadening of the peaks due to the combine effect of crystallites size and strain is given by;

(9) $$\beta_{T}=\beta_{D}+\beta_{Ɛ}$$

Where $\beta_{T}$ is total broadening; $\beta_{D}$ is broadening due to crystallites size and $\beta_{Ɛ}$ is broadening due to strain.

By adding equations (5), (7) and then rearranging into [53];

(10) $$\beta_{T\;}\;\cos\;\theta =\frac{K\lambda }{D}+4\;Ɛ\;\sin\;Ɵ$$

**Fig. 4:**
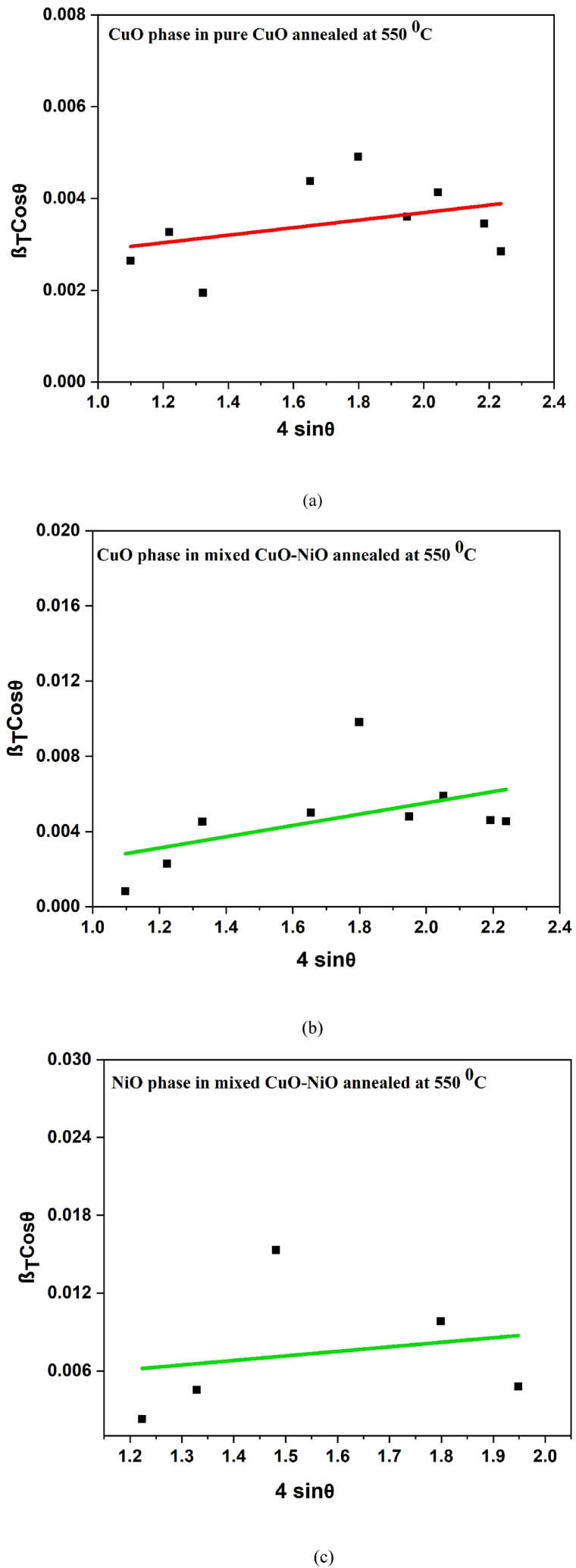

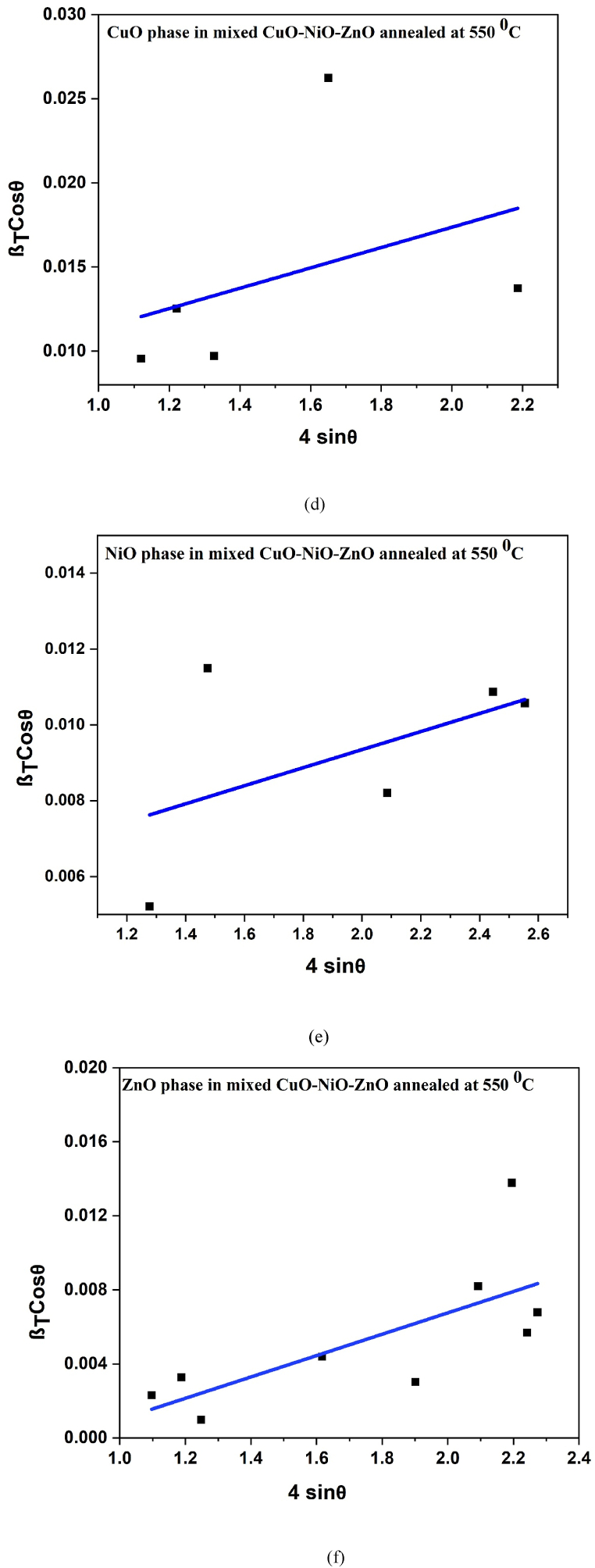
The Williamson- Hall plots (a) for CuO phase in pure CuO, (b) for CuO phase in mixed CuO–NiO, (c) for NiO phase in mixed CuO–NiO, (d) for CuO phase in mixed CuO–NiO–ZnO, (e) for NiO phase in mixed CuO–NiO–ZnO, and (f) for ZnO phase in mixed CuO–NiO–ZnO nano-powders after annealing at 550 °C.

**Fig. 5:**
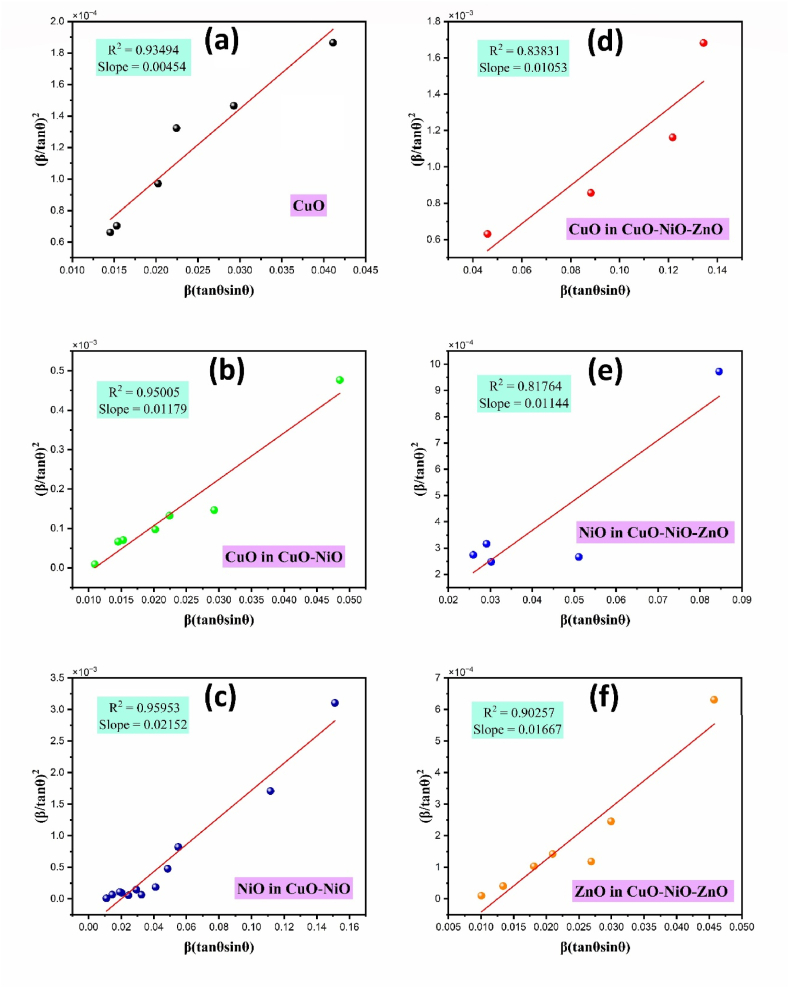
The Halder-Wagner plots for (a) CuO phase in pure CuO, (b) CuO phase in mixed CuO–NiO, (c) NiO phase in mixed CuO–NiO, (d) CuO phase in mixed CuO–NiO–ZnO, (e) NiO phase in mixed CuO–NiO–ZnO, and (f) ZnO phase in mixed CuO–NiO–ZnO nano-powders after annealing at 550 °C.

This is Williamson-Hall equation. By plotting $\beta_{T}\;\cos\;\theta$ against 4sinƟ for CuO, NiO and ZnO as shown in Fig. 4, the average crystallite size was obtained from the y-intercept and the RMS microstrain from the slope. The lattice microstrain values obtained from equation (8) are given in Table 1. The crystallite sizes calculated from the three methods according to equations (6), (7), (10) are almost the same for the CuO and NiO phases but the Williamson-Hall gave a slightly larger value than the Scherer method for the CuO phase. The difference in the values obtained from Scherer and W–H plot analysis shown in Fig. 4 (a), (b), (c), (d), (e) and (f); could be due to the difference in averaging the particle size distribution [53]. The higher lattice microstrain of CuO phase in presence of NiO and ZnO phases suggests the increase in lattice distortions, grain boundaries, and defects. The introduction of NiO and ZnO phases may have induced lattice distortions, leading to higher microstrain values. These distortions can be attributed to differences in lattice parameters between the constituent phases. Similarly, the Halder-Wagner (H–W) plots [54] for CuO phase in pure CuO; CuO phase in mixed CuO–NiO; NiO phase in mixed CuO–NiO; CuO phase in mixed CuO–NiO–ZnO; NiO phase in mixed CuO–NiO–ZnO, and ZnO phase in mixed CuO–NiO–ZnO nano-powders were studied as shown in Fig. 5 (a), (b), (c), (d), (e) and (f), respectively. It was found well fit with the data and crystallite sizes of all phases were in nano size. The crystallite sizes values are given in Table 1.

**Table 1:** The crystallite sizes, lattice microstrain, dislocation density, X-ray density, and specific surface area values determined from XRD profile analysis of CuO, CuO–NiO, and CuO–NiO–ZnO nano-powders.

Metal oxide nanoparticles	Oxide Phase	Crystallite size D (nm)				Microstrain (Ɛ $\times 10^{-3}$)	Dislocation density (***δ***_*np*_$\times 10^{-3}\;{nm}^{-2})$	X-ray density ($\rho_{x})$$g/{\text{cm}}^{3}$	Specific surface area (m^2^/g)
		Scherrer formula	Modified Scherrer equation	W–H plot	H–W plot				
CuO	CuO	43	49.18	66.05	30.54	2.074	0.663	6.48	17
CuO–NiO	CuO	46	76.25	46.22	11.76	2.665	1.431	6.51	16
	NiO	8.02	4.05	5.01	6.44	10.548	10.976	3.89	271
CuO–NiO–ZnO	CuO	11.05	12.74	26.21	13.17	9.654	12.678	6.53	55
	NiO	16.29	20.56	30.27	12.12	4.879	4.748	6.80	39
	ZnO	43.69	55.07	29.01	8.32	2.853	2.201	5.72	24

The diffraction peaks at 2 θ = 31.8613°, 35.6089°, 38.7952°, 48.8447°, 53.452°, 58.2902°, 61.6943°, 66.4647° and 68.0757° which corresponds to (110), ($\bar{1}$ 11), (111), ($\bar{2}$ 02), (020), (202) ($\bar{1}$ 13), (220), ($\bar{3}$ 11) and (220) Miller planes and the diffraction peaks at 2θ = 19.301°, 33.161°, 43.274°, 52.243°, 59.242° and 62.921° which corresponds to (001), (100), (10 $\bar{1}$), (200), (10 $\bar{2}$), (110), and (111) Miller planes were selected for calculating the average crystallite size for CuO and NiO phases in CuO–NiO mixed oxide NPs, respectively. The diffraction peaks at 2 θ = 32.531°, 35.466°, 35.556°, 38.751°, 38.965°, 48.751° and 66.279° which corresponds to (110), (002), ($\bar{1}$ 11), (111), (200), ($\bar{2}$ 02) and ($\bar{3}11$) Miller planes; the diffraction peaks at 2 θ = 37.237°, 43.265°, 62.846°, 75.373° and 79.364° which correspond to (111), (020), (022), (131) and (222) Miller planes and the diffraction peaks at 2 θ = 31.847°, 34.55°, 36.354°, 47.694°, 56.745°, 63.087°, 66.558°, 68.161° and 69.284° which correspond to (100), (002), (101), (102), (110), (103), (200), (112), and (201) Miller planes were selected for calculating the crystallite size for CuO, NiO and ZnO phases in CuO–NiO–ZnO mixed oxide NPs, respectively. The average of these measurements was reported as crystallite size for each phase. The crystallite sizes for the synthesized metal oxide phases calcined at 550 °C are listed in Table 1. It was observed that CuO phases had almost the same crystallite sizes in pure CuO and mixed CuO–NiO, but in CuO–NiO–ZnO mixed oxide nano-powder CuO phase showed the smaller crystallite size suggesting a higher degree of nanostructuring. The decrease of crystallite sizes of CuO phase and the increase of NiO phase in CuO–NiO–ZnO, indicating a possible influence of ZnO on the crystal growth [27]. This observation could be related to the formation of new phases or solid solution structures due to the interaction between the different metal oxides.

#### 3.2.5 Dislocation density and crystallinity

The crystallite sizes of different phases present in CuO, CuO–NiO, and CuO–NiO–ZnO nano-powders provide information about the degree of crystallinity as well as disclose the potentiality for improved material properties as it reflects the size of the individual crystalline domains within the nanopowders. Crystallinity refers to the degree of long-range structural order in a material and the degree of crystallinity strongly affects mechanical and thermal properties. The more crystalline, the more hardness and density. The crystallinity of the nanoparticles controlled with different parameters. The degree of crystallinity decreased significantly with decreasing NPs diameter. Based on the area of crystalline peaks and area of all peaks (crystalline + amorphous) the degree of crystallinity can be obtained using the following expressions;

(11) $$\text{Crystallinity}=\frac{\text{area}\;\text{of}\;\text{crystalline}\;\text{peaks}\;}{\text{area}\;\text{of}\;\text{all}\;\text{peaks}\left(\;\text{crystalline}+\text{amorphous}\right)}\times 100\%$$

From Table 3 it is shown that all the synthesized nanopowder were formed with high degree crystallinity. As dislocations interact with the crystallinity and microstructure of the material and illustrate the size of the crystal defect or the degree of crystallinity of the nanoparticles profile. It is needed to calculate the dislocation density value (δ). Based on the average crystallite size values as calculated, the dislocation density values of the synthesized nanoparticles can be determined using equation [55];

(12) $$\delta_{np}=1/D^{2}$$

**Table 2:** Lattice parameters of the synthesized nano-powders of CuO, CuO–NiO, and CuO–NiO–ZnO nano-powders from their respective XRD profile analysis.

METAL OXIDE NANOPARTICLES	OXIDE PHASE	CRYSTAL SYSTEM	CRYSTAL CLASS	a(Å)	b(Å)	c(Å)	VOLUME(Å^3^)
CuO	CuO	monoclinic	Domatic	4.693	3.428	5.137	81.50
CuO–NiO	CuO	monoclinic	Prismatic	4.684	3.423	5.129	81.10
	NiO	hexagonal	Ditrigonal scalenohedral	3.1170	3.1170	4.595	38.66
CuO–NiO–ZnO	CuO	monoclinic	Prismatic	4.683	3.420	5.125	80.91
	NiO	cubic	Hexoctahedral	4.179	4.179	4.179	72.98
	ZnO	hexagonal	Dihexagonal Pyramidal	3.242	3.242	5.188	47.22

**Table 3:** Average diameter (SEM), degree of crystallinity (XRD) and elemental composition (EDX) of CuO, CuO–NiO and CuO–NiO–ZnO nano-powders.

Metal oxide nanoparticles	Degree of crystallinity (%)	Average diameter (NM)	Copper (%)	Nickel (%)	Zinc (%)	Oxygen (%)
CUO	81.44	83.707 ± 1.994	50.38	0	0	49.62
CUO-NIO	73.06	15.514 ± 0.157	29.87	3.84	0	66.29
CUO-NIO-ZNO	71.32	56.615 ± 4.165	3.38	26.03	16.66	53.93

Table 3, Table 1 present the calculated values of the degree of crystallinity and dislocation density, respectively, derived from equations (11) and (12) for the as-synthesized nanopowders. From Table 1 it is observed that, mixing of CuO with NiO followed by calcination at 550 °C led to a slight increase in dislocation density of CuO phase but mixing of CuO with NiO and ZnO followed by calcination at 550 °C led to a great increase in dislocation density of CuO phase. It is because the smaller crystallite size of CuO phase in CuO–NiO–ZnO mixed oxide NPs lead to increase in surface atom that is an energetic surface [55]. To recapitulate, small dislocation density values of all the synthesized nano-powder indicate the high degree of crystallinity. The observed dislocation density values explain the mechanical and thermal history of the nano-powders during synthesis and post-processing steps. Which may impact material properties such as mechanical strength and electrical conductivity.

#### 3.2.6 Lattice constant, X-ray density and specific surface area

The lattice constant (or lattice parameter) as a function of temperature and composition indicates the constant distance between unit cells in a crystal lattice. Also provide information for the modeling of microstructure growth and packing efficiency. The lattice parameters a, b, c are the lengths of the unit cell in three dimensions and "α, β, γ," their mutual angles. The crystal system of as-synthesized pure CuO NPs is monoclinic belonging to domatic (base-centered) crystal class with space group (C 1 c 1) and space group number 9. The crystal system of as-synthesized mixed CuO–NiO NPs has two phases as shown in Table-2. Where, CuO phase shows monoclinic belonging to prismatic (base-centered) crystal class with space group (C 1 2/c 1) and space group number 15 and NiO phase shows hexagonal belonging to Ditrigonal scalenohedral (primitive) crystal class with space group (P $\bar{3}$ m1) and space group number 164. In CuO–NiO–ZnO there are three phases as shown in Table-2. Where, CuO phase shows monoclinic belonging to prismatic (base-centered) crystal class with space group (C 1 2/c 1) and space group number 15; NiO phase shows cubic belonging to Hexoctahedral (face-centered) crystal class with space group (Fm $\bar{3}$ m) and space group number 225 and ZnO phase shows hexagonal belonging to Dihexagonal Pyramidal (primitive) crystal class with space group (*P*6_3_*mc*) and space group number 186. All the lattice constant (or lattice parameter) as shown in Table-2 explain the nano sized structure and microstructure growth and packing efficiency of the as synthesized nanopowders. Whereas with the higher value of the lattice constants enhanced antibacterial activity is reported [56]. The obtained lattice parameters for CuO nanopowder are in good agreement. This consistency suggests that the synthesis method employed in this study has successfully produced CuO NPs with a well-defined crystal structure. Comparing the lattice parameters of CuO and NiO in the CuO–NiO nanopowders with those of the respective pure phases it is found the presence of multiple phases. These lattice parameters were also used to estimate the relative composition of CuO and NiO phases within the nanopowders. The lattice parameters of CuO, NiO, and ZnO in the CuO–NiO–ZnO nanopowders assess the degree of crystallinity and phase composition. Where no significant deviations in the lattice parameters from the values of pure CuO, NiO, and ZnO phases were observed.

With the help of unit cell parameters, a, b & c the X-ray density [57] has been calculated using following standard relations shown in eq.;

(13) $$\rho_{x}=\frac{Z\;M}{N_{A}\;a^{3}}$$

Where N_A_ is Avogadro's number (6.023 × 10^23^ mol^−1^), M is the molecular weight, Z is the number of formula unit present in a unit cell and (a) is lattice constant.

The specific surface area (m^2^/g) of the annealed nanocrystalline powders were calculated with the help of X-ray density ($\rho_{x}$) and average crystalline size (D) from the following expression [58];

(14) $$S=\frac{6000}{D\times \rho_{x}}$$

Table 1 displays the calculated X-ray density and specific surface area, determined using equations (13), (14) respectively. The observed higher specific surface area of CuO (55 m^2^/g) phase in CuO–NiO–ZnO NPs, may be advantageous for applications such as catalysis. X-ray density of the synthesized nanopowders explain the formation of nano structure which is sensitive to crystal structure. It is shown that X-ray density values for CuO (∼6.50 g/cm^3^) phases in CuO, CuO–NiO, and CuO–NiO–ZnO nano-powders remain almost same. Which indicates the level of porosity and densification achieved positively during synthesis [59].

### 3.3 Elemental analysis (EDX) and surface morphology

The Energy dispersive x-ray (EDX) analysis was carried out for the elemental composition analysis and purity of the synthesized CuO, CuO–NiO and CuO–NiO–ZnO nano-powders by atom %. In this study it was found that CuO NPs calcined at 550 °C contains only Copper & Oxygen, CuO–NiO NPs calcined at 550 °C contains only Copper, and Nickel & Oxygen and CuO–NiO–ZnO NPs calcined at 550 °C contains only Copper, Nickel, and Zinc & Oxygen as shown in Fig. 8 (a), (b), and (c). From the results (Table 3) it is found that the presence of Cu element significantly decreases from 50.38 % (CuO) to 3.38 % (CuO–NiO–ZnO). But in case of Ni element, it is increased from 3.84 % (CuO–NiO) to 26.03 % (CuO–NiO–ZnO). This discrepancy highlights the complex interplay of various factors influencing nanoparticle synthesis and underscores the importance of careful characterization and optimization of synthesis conditions. The decrease in Cu content can result from various factors, including isomorphous substitution of Cu with other metal cations, phase segregation during synthesis, or preferential growth of alternative phases [60]. It suggests that the addition of NiO and ZnO has displaced or replaced a significant portion of the copper oxide phase. This decrease suggests that a significant portion of the copper oxide phase has been displaced or replaced by other components, such as NiO and ZnO, resulting in a shift in the material's composition. Conversely, the increase in the presence of Ni from 3.84 % in CuO–NiO nanoparticles to 26.03 % in CuO–NiO–ZnO nanoparticles signifies a notable enrichment of nickel within the material. This increase indicates that the incorporation of NiO within the material has been enhanced in the presence of ZnO, leading to a higher proportion of nickel oxide in the final composition. The increase in Ni content may be attributed to the preferential growth or stabilization of NiO phases in the presence of ZnO [61], promoting the retention of nickel within the material matrix.

**Fig. 6:**
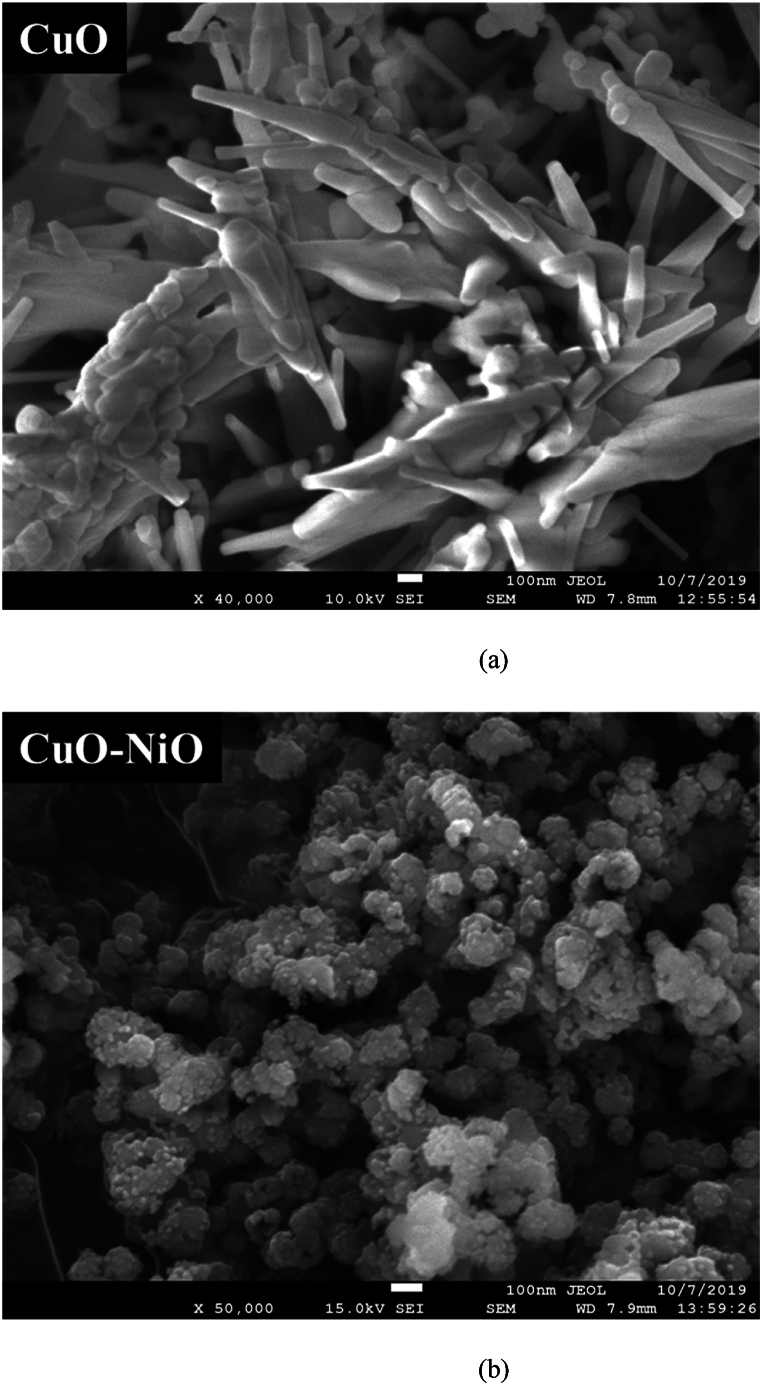

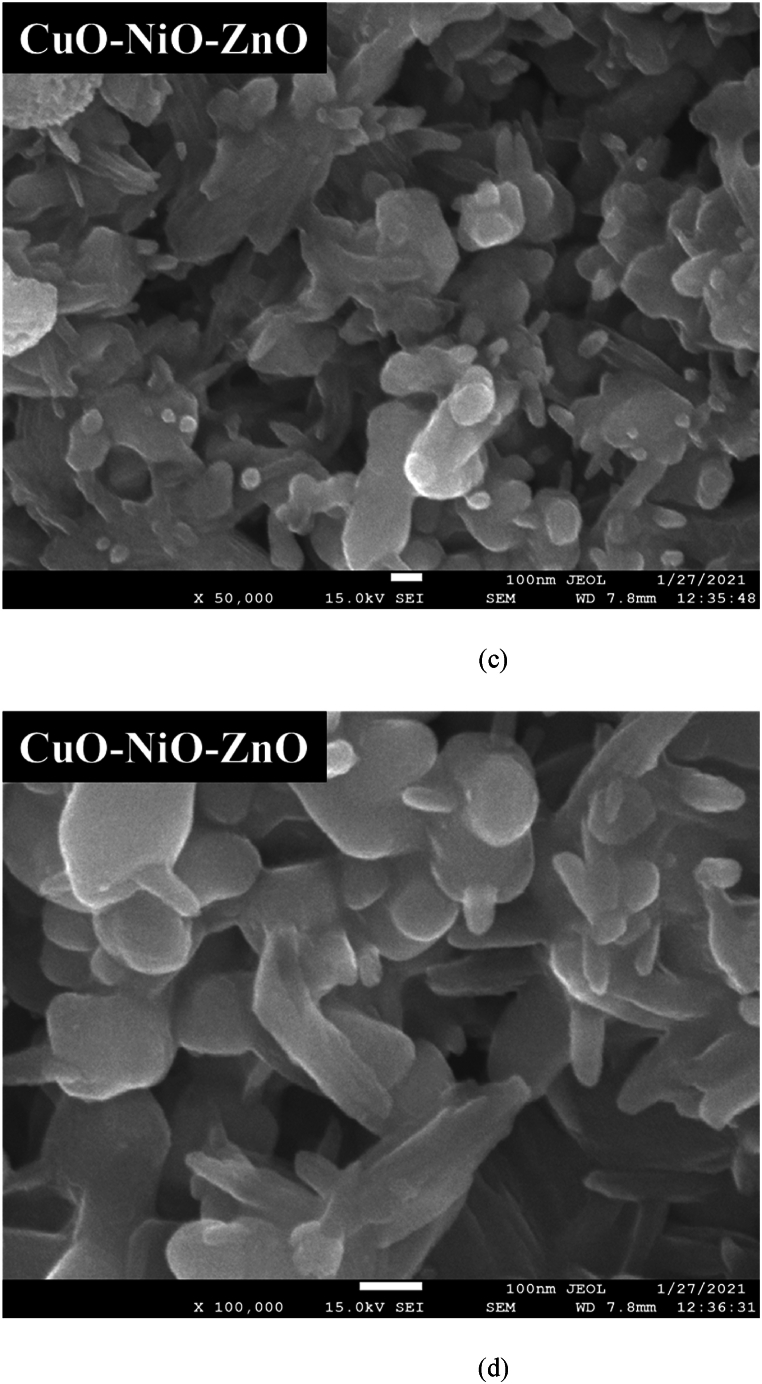
SEM images denoted as (a) for CuO, (b) for CuO–NiO, and (c) & (d) for CuO–NiO–ZnO nano-powders after annealing at 550 °C.

**Fig. 7:**
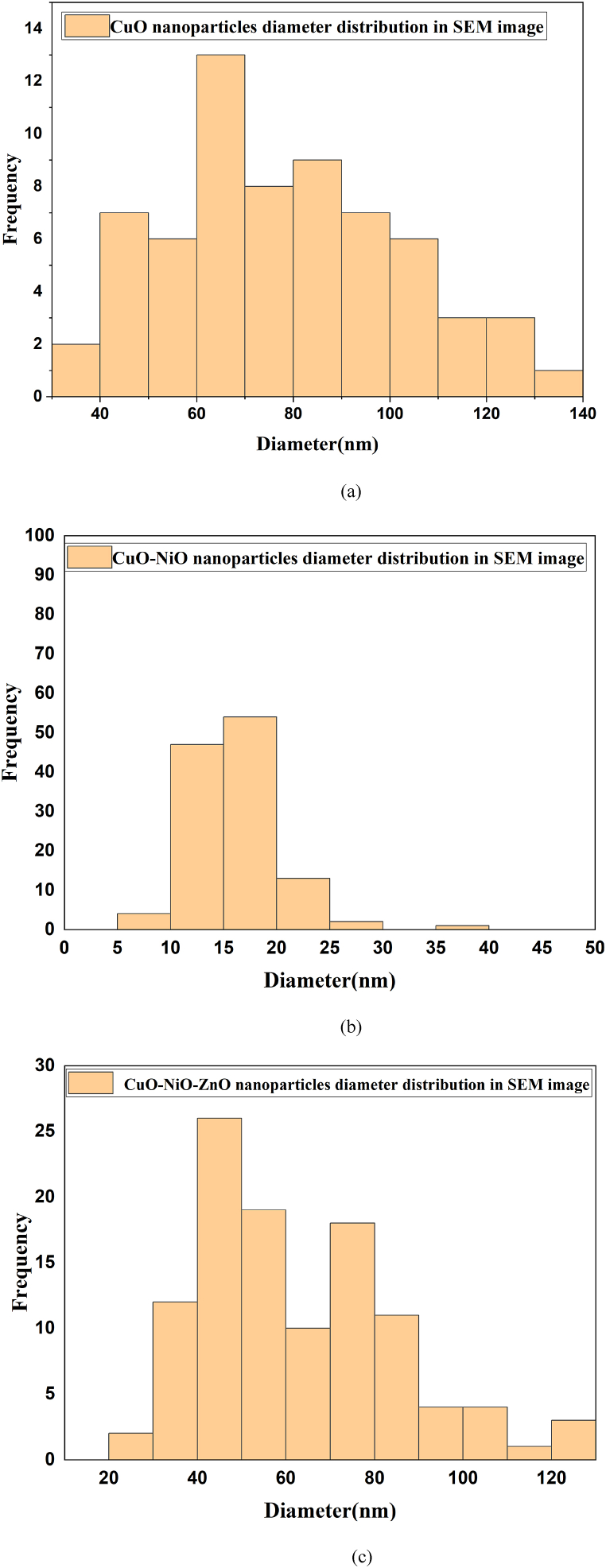
Particles diameter distribution according to SEM images denoted as (a) for CuO, (b) for CuO–NiO, and (c) for CuO–NiO–ZnO nano-powders after annealing at 550 °C.

The morphologies of the synthesized CuO, CuO–NiO and CuO–NiO–ZnO nano-powders were analyzed by SEM with different magnification. The SEM image of the CuO, CuO–NiO and CuO–NiO–ZnO mixed oxide NPs is presented in Fig. 6 denoted as (a) for CuO, (b) for CuO–NiO, and (c) & (d) for CuO–NiO–ZnO. The observation of a structural transition from rod-like morphology in CuO nanoparticles to a flower-like morphology in CuO–NiO mixed nanoparticles, as depicted in the SEM images. The formation of rod-like structures in CuO nanoparticles and flower-like structures in CuO–NiO mixed nanoparticles can be influenced by the kinetics of crystal growth and aggregation during the synthesis process [62]. In the case of CuO nanoparticles, the growth and aggregation of crystals may favor the elongation along specific crystallographic directions, leading to the formation of rod-like structures. However, the introduction of NiO into the system alter the crystal growth kinetics and promote the nucleation and growth of crystals in different directions, resulting in the formation of flower-like structures with multiple branches. On the other hand, the presence of NiO in the mixed nanoparticles can modify the surface energy and promote the formation of specific crystal sides that lead to the development of flower-like structures with branching patterns [50]. Reaction temperature, precursor concentration, solvent composition, and reaction time can affect the nucleation and growth rates, as well as the crystal phase and morphology of the nanoparticles [63]. Understanding these factors is essential for modifying the morphology and properties of metal oxide nanoparticles. In the SEM images, it was observed that all the synthesized nano-powders exhibited a relatively uniform and small average diameter. The average diameter of the synthesized NPs was measured from SEM images with the help of ImageJ software. Particles diameter distribution according to SEM images denoted as (a) for CuO, (b) for CuO–NiO, and (c) for CuO–NiO–ZnO are shown in Fig. 7 and the calculated values of average diameter are given in Table 3. It was found well agreement with the average crystallite size (D) obtained from XRD data analysis. From the images rod like shape of pure CuO phase was observed. But in the case of CuO–NiO and CuO–NiO–ZnO nano-powders, the SEM images revealed a more complex morphology due to the presence of multiple phases. The morphology of the NPs, whether they are agglomerated or dispersed, can be significant for applications involving material reactivity and surface interactions.

**Fig. 8:**
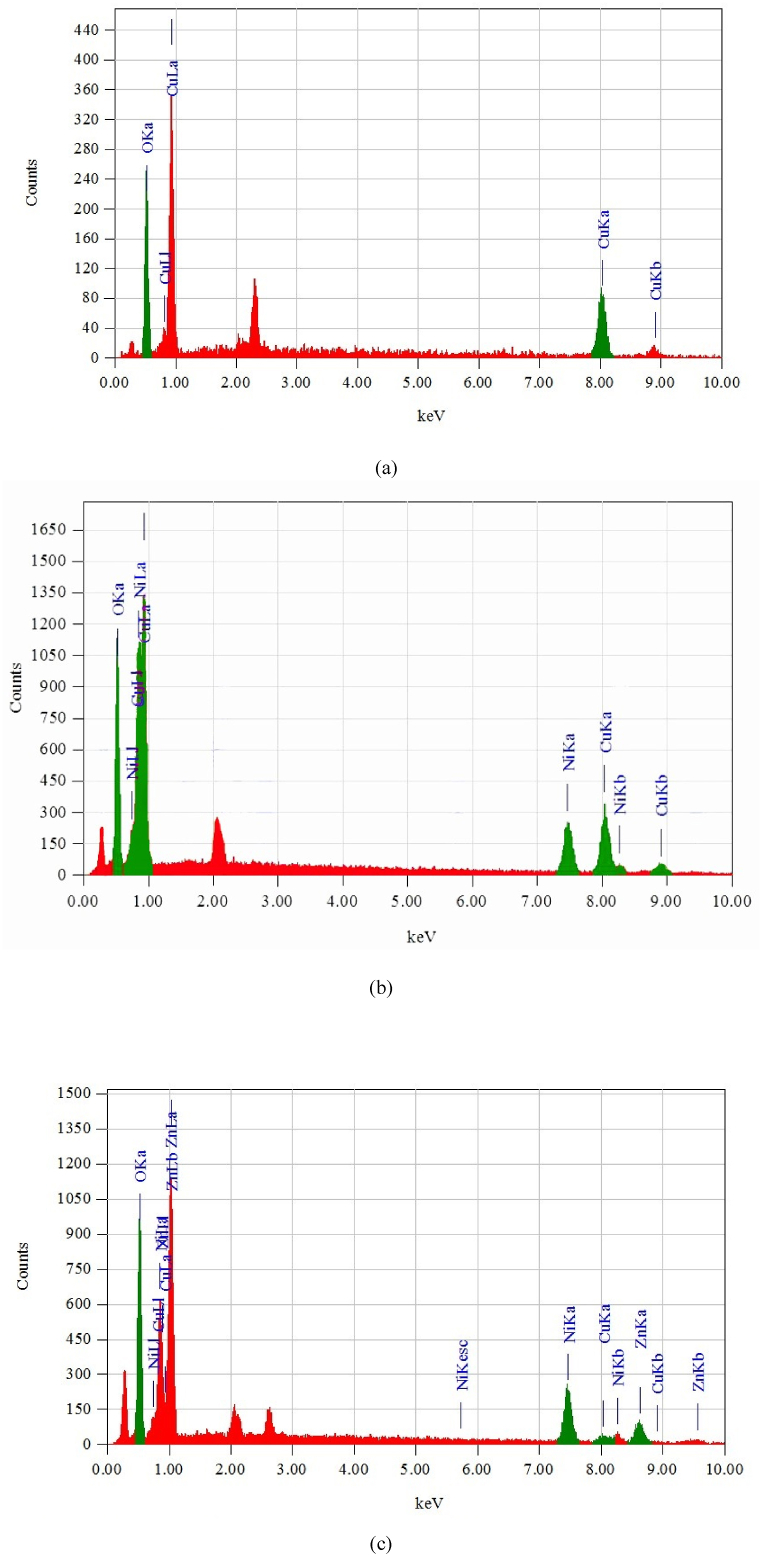
EDX images denoted as (a), (b) and (c) for CuO, CuO–NiO, and CuO–NiO–ZnO nano-powders, respectively.

## 4 Conclusion

CuO, CuO–NiO and CuO–NiO–ZnO nano-powders were successfully prepared via precipitation and co-precipitation method. In fact, the precipitation and co-precipitation preparation of these oxide nanopowders was composed of two stages: the formation of meta-stable copper, copper-nickel and copper-nickel-zinc precursor precipitate and the subsequent transformation of this precipitate to nano CuO, CuO–NiO and CuO–NiO–ZnO by calcined at 550 °C. Therefore, various parameters like solution concentration, stirring time and calcination temperature have to be controlled as they play substantial roles in the preparation of nanoscale powder. Comparison of XRD's of metal oxides with JCPDS and COD No. confirmed the presence of single and multi-phases i.e.; the formed metal oxides were CuO, CuO–NiO and CuO–NiO–ZnO. Average crystallite sizes calculated from Debye-Scherrer equation, modified Debye-Scherrer equation, Halder-Wagner equation and Williamson-Hall equation conformed nano size of the synthesized powders. High crystallinity and nanostructure of the synthesized NPs were conformed from small dislocation density and microstrain. The Miller indices were matched with base-centered monoclinic structure of CuO, monoclinic with primitive hexagonal structure of CuO and NiO phases in mixed CuO–NiO and base-centered monoclinic structure of CuO phase, faced centered cubic structure of NiO and primitive hexagonal structure of ZnO phase in mixed CuO–NiO–ZnO. The vibrational frequency was confirmed by FT-IR analysis and it was found that the vibration peaks for CuO, NiO and ZnO occurring in the region below 600 cm^−1^. SEM results were in well agreement with the size distribution and average diameter of CuO, CuO–NiO and CuO–NiO–ZnO NPs measured by XRD. The presence of copper, nickel, zinc, and oxygen in CuO, CuO–NiO and CuO–NiO–ZnO was confirmed by EDX analysis. This study explains single and multiphase mixed oxide nanoparticles of copper, nickel, and zinc. These findings are essential for tailoring the properties of these materials for various engineering applications, including catalysis, sensors, and energy storage devices. Further research and characterization are necessary to fully understand the core mechanisms influencing these parameters and to optimize material performance.

## Data availability statement

The dataset associated with this study is not currently stored in a public data repository. Data will be made available on request.

## CRediT authorship contribution statement

**Md. Jasim Uddin:** Writing – review & editing, Writing – original draft, Software, Formal analysis, Data curation. **Mst. Sarmina Yeasmin:** Writing – review & editing, Validation. **Ali Ahsan Muzahid:** Data curation. **Md. Mahmudur Rahman:** Investigation. **G.M. Masud Rana:** Resources. **Tahmina Akter Chowdhury:** Visualization. **Md. Al-Amin:** Formal analysis. **Md. Kazi Wakib:** Software, Methodology. **Sayeda Halima Begum:** Writing – review & editing, Supervision, Conceptualization.

## Declaration of competing interest

All authors have read and approved the final manuscript for publication. We collectively affirm that we have no known financial, non-financial, or research-based conflicts of interest that could potentially influence the objectivity or integrity of the research presented in this manuscript.
